# Knowledge, attitudes and practices related to antimicrobial use and resistance among fish farmers in Dar es Salaam, Tanzania

**DOI:** 10.1371/journal.pone.0335862

**Published:** 2025-11-11

**Authors:** Zainabu Hamisi Kilusungu, Zuhura Idd Kimera, Wilson Nandolo, Peter Kunambi, Fauster Mgaya, Mecky Isaac Matee, Daud Kassam

**Affiliations:** 1 Department of Aquaculture and Fisheries Science, Africa Centre of Excellence in Aquaculture and Fisheries (AquaFish), Lilongwe University of Agriculture and Natural Resources, Lilongwe, Malawi; 2 Department of Environmental and Occupational Health, Muhimbili University of Health and Allied Sciences, Dar es Salaam, Tanzania; 3 Department of Animal Science, Lilongwe University of Agriculture and Natural Resources, Lilongwe, Malawi; 4 Department of Microbiology and Immunology, School of Medicine, Muhimbili University of Health and Allied Sciences, Dar es Salaam, Tanzania; 5 SACIDS Foundation for One Health (SACIDS), Sokoine University of Agriculture (SUA), Morogoro, Tanzania; CSSRI: Central Soil Salinity Research Institute, INDIA

## Abstract

This study was conducted in Dar es Salaam, Tanzania, the major commercial city of Tanzania, to assess the knowledge, attitudes, and practices (KAP) of fish farmers on antimicrobial use (AMU) in aquaculture. This was a cross-sectional study conducted between March and June 2023, involving total of 60 fish farmers. Data were collected electronically using the Afya-Data application and analysed with SPSS Version 26.0. The findings showed that most farmers were male (71.7%), over 50 years of age (51.7%), and college-educated (38.3%). However, 80% had not received formal aquaculture training, and 50% lacked access to aquaculture extension services. While 80% of the respondents were aware of antibiotics and 93% were familiar with antimicrobial resistance (AMR), only 35% demonstrated a positive attitude towards the use of antimicrobial agents. Although no farmer reported direct antibiotic use, potentially risky practices were noted. These included the use of treated manure for pond fertilization in 38.3% of farms and irregular pond drainage into the environment practices that can contribute to the spread of AMR beyond aquaculture settings. To address these gaps, we recommend strengthening aquaculture extension services with a focus on improved pond management practices, establishing a functional fish disease surveillance system, and enhancing collaborative research among government, academic, and research institutions. The Department of Aquaculture Extension should develop cost-effective farmer-to-farmer extension models. To improve access to information, we also propose forming small cooperative groups under the Aquaculture Association of Tanzania (AAT). Moreover, the Ministry of Livestock and Fisheries (MLF) should develop and implement clear guidelines for monitoring AMU, AMR, and fish diseases, both existing and emerging.

## Introduction

On a global scale, the use of antimicrobials in aquaculture is well recognized as a significant factor contributing to the development of antimicrobial resistance (AMR) [1–6], and has been associated with negative economic and social repercussions [1]. In Africa, the problem of AMR in livestock farming is compounded by i) weak regulations and surveillance systems [7], ii) limited access to appropriate antimicrobial therapy [7]; and iii) inadequate knowledge of AMU and AMR [8]. In addition, farm owners tend to stock drugs and treat animals on their own [9].

Various studies have documented substantial variations in the amounts of antimicrobials used in different aquaculture production systems, highlighting variances in the factors that influence these quantities [10]. Nevertheless, there is a lack of comprehensive documentation regarding the extent and trends of antimicrobial usage in aquaculture throughout Sub-Saharan Africa [8,11]. This situation restricts the implementation of specific treatments and regulations that promote responsible use of antimicrobials [11].

Tanzania is experiencing rapid growth in the aquaculture industry, creating employment and investment opportunities [12]. Freshwater fish farming dominates the industry where by mostly are small-scale farmers engaging in both extensive and semi-intensive fish farming [13]. The integrated fish farming system, such as the fish-poultry and fish-horticulture system is common because it produces high yields with low input, while the fish receives limited, if any supplementary feed [14].

Due to the increasing popularity of aquaculture industry in the nation, the use of antimicrobial agents meant for livestock, especially poultry has been frequently observed [15]. In addition, the application of cattle and poultry dung from treated animals to fertilize ponds is considered an indirect means of introducing antimicrobial agents, but is a common practice [15]. Antimicrobial agents, especially antibiotics, such as tetracycline (chlortetracycline and oxytetracycline), colistin, sulphonamides, and neomycin are added to livestock feeds as supplements [15]. Draining of fish ponds to water vegetables, which is very common, has been associated with the continuous release of pond water into the environment [16], promoting the rapid increase of antibiotic-resistant bacteria (ARB) and antibiotic-resistance genes (ARGs) in soils [17]. Subsequently, resistance can spread in various ways, to reach humans and animals through contamination of the environment [18].

Considering the growing economic importance of aquaculture products in Tanzania urgent measures are required to prevent AMR. Of particular importance is the determination of factors influencing AMU behaviours and practices of fish farmers [19]. This is key in laying the foundation for behavior modification that is necessary when devising complex interventions or methods to achieve successful and long-lasting changes in AMU behavior [19].

Different studies that explore the knowledge, attitudes, and opinions of farmers regarding antimicrobial resistance and the usage of antimicrobials have yielded diverse and contradictory results across different nations [20]. These observations emphasize the need to conduct local studies that may assist in developing customized intervention methods to enhance judicious antimicrobial use. The present study conducted a targeted evaluation of the knowledge, attitudes, and practices (KAP) of aquaculture farmers in Dar es Salaam, Tanzania towards AMU and AMR. The study sought to gather vital information required for an evidence-based approach that incorporates behavior change theory, which is necessary for devising complex interventions or strategies to effectively and consistently induce successful and lasting changes in individuals’ behavior towards AMU. This study hypothesized that developing evidence-based effective techniques to encourage and maintain changes in behavior is crucial in reducing reckless antimicrobial usage in aquaculture, thus limiting the contribution of the industry to the global health threat of AMR.

## Materials and methods

### Study area and design

This was a cross-sectional study conducted in Dar es Salaam Regions in Tanzania, between March and June 2023. Dar es Salaam is the major commercial city of Tanzania, with a population of 5,383,728 (8.7% of all Tanzanians) [21] and has the highest demand for fish in the country. The region comprises five districts namely Ilala, Kinondoni, Temeke, Ubungo, and Kigamboni. The reported number of registered fish farmers from Aquaculture zonal office is 47 of which 32 farmers farm Tilapia and the rest farm African catfish, ornamental fish, and crabs All fish farmers within the study area were selected to participate in this study, including others who were not officially registered making a total of 60 fish farmers.

### Data collection

A comprehensive questionnaire was created, consisting of closed-ended, open-ended, and multiple-choice questions, to evaluate a fish farmer’s knowledge, attitude, practice, and farm administration in relation to AMU, AMR, and the presence of antimicrobial residues. The collected data encompassed socio-demographic attributes such as age, gender, occupation, village, workplace, and residence. It also included information on the knowledge on antimicrobials, including their definition, roles, and types. Additionally, the data covered practices related to antimicrobial usage, such as preferences, frequency of use, sources of acquisition, specific illnesses treated, adherence to treatment regimes, and self-medication practices. Additionally the study evaluated the level of awareness and understanding regarding antimicrobial resistance (AMR). Apart from using dichotomous responses for certain questions, the participants’ knowledge and attitudes towards antimicrobials, their uses, burden, actions, and roles in addressing antimicrobial resistance (AMR) were determined using a five-point Likert scale. The scale included options such as ‘strongly agree’, ‘agree’, ‘uncertain’, ‘disagree’, and ‘strongly disagree’.

The participants questioned in Swahili, and their responses captured exactly as spoken and transcribed into the coded questionnaire on a mobile phone or tablet. Piloting of the questionnaires was done among 10 farmers from Mkuranga district in Pwani region and necessary adjustments were done before conducting the actual interviews.

### Data management and analysis

The data were gathered electronically using the AfyaData program version 1.0, a mobile data application [22] and were instantly transmitted to a server situated at Sokoine University of Agriculture (SUA) in Morogoro, Tanzania. The data, in the form of an Excel spreadsheet, were extracted from the server, coded, and imported into the IBM SPSS Version 26.0 [23] for data analysis. Descriptive statistics was used to provide overview of the socio-demographic features and aquaculture farming practices of the farmers. Categorical variables were presented as frequencies, while numerical variables were presented as median (IQR).

A composite variable was generated by combining the knowledge questions. Participants who answered 75% of the questions correctly were classified as having adequate knowledge of antimicrobial use. Similarly, the questions in the attitude scale were combined into a composite variable, and participants whose responses indicated a positive attitude towards antimicrobial use in 80% of the questions were classified as having a positive attitude. The questions in the practice scale were also combined into a single composite variable, and participants who demonstrated appropriate practices in 75% of the questions were regarded as having good practices.

The chi-square test was employed to evaluate the association between socio-demographic characteristics, aquaculture management, and knowledge, attitude, and practices. The Spearman rank correlation coefficients (rho) were employed to evaluate the association between farmers’ knowledge, attitude, and practices concerning antibiotic use.

### Ethical considerations

The study obtained Ethical approval from National Institute for Medical Research (NIMR/HQ/R. 8a/Vol.IX/4225) and Tanzania Fisheries Research Institute (TAFIRI/HQ/RES.CLEARANCE/108). Written consent form was provided to the participants to sign before commencement of the interview after being briefed about the objectives of the study. Participants were given an opportunity to withdraw from the study at any time without prejudice.

## Results

### Sociodemographic characteristics

A total of 60 farmers were enrolled, the majority of whom were males (71.7%), aged more than 50 years (51.7%), owned individual farms (65%), and (38.3%) were university graduates (Table 1). Additionally, about 63.4% of the farmers had been in the aquaculture business for less than 5 years, and only 13.3% primarily depended on fish farming as a source of income.

**Table 1:** Sociodemographic characteristics of the study participants (n = 60).

Variable	Frequency (n)	Percent (%)
**Age of the respondent**		
21–30	9	15.0
31–40	7	11.7
41–50	13	21.7
≥ 50	31	51.7
**Gender of the owner**		
Male	43	71.7
Female	17	28.3
**Residence (Districts)**		
Kigamboni	8	13.3
Kinondoni	15	25.0
Temeke	16	26.7
Ubungo	10	16.7
Ilala	11	18.3
**Ownership of the farm**		
Company	6	10.0
Family	12	20.0
Group	3	5.0
Individual	39	65.0
**Level of education**		
No formal education	1	1.7
Primary	14	23.3
Secondary	15	25.0
Diploma	7	11.7
University	23	38.3
**Working experience in Aquaculture (years)**		
< 1	4	6.7
1–5	34	56.7
6–10	12	20.0
11–15	7	11.7
>15	3	5.0
**Primary occupation of the owner**		
Artisan	1	1.7
Business	17	28.3
Civil servant	10	16.7
Fish farmer	8	13.3
Livestock keeper	8	13.3
Agriculture	7	11.7
Retired	9	15.0

### Mapping of aquaculture activities

The majority of the farms owned less than one hectare (61.7%), and most of the farmed fish were tilapia followed by catfish (Table 2). The dominant farming practices was semi-intensive, monoculture, with a production cycle of between 6 and 8 months. Pond fertilization was observed in 38.3% of the farms, and most feeds were bought from shops.

**Table 2:** Mapping of aquaculture activities.

Variables	Frequency (n)	Percent (n)
**Size of the farm**		
Less than 1 Ha	37	61.7
1 to 5 Ha	23	38.3
**Types of fish species cultured**		
Tilapia	30	50
Cat fish	6	10
Tilapia and Catfish	22	36.7
Others (Mkunga, Prawns)	2	3.4
**Level of management**		
Extension	6	10
Intensive	7	11.7
Semi intensive	47	78.3
**Culture system**		
Monoculture	47	78.3
Polyculture	13	21.7
**Production cycle**		
Less than 6 months	2	3.3
6 to 8 months	31	51.7
More than 8 months	27	45.0
**Pond fertilization**		
Yes	23	38.3
No	37	61.7
**Source of formulated feed**		
Home made	18	30
Not using formulated feed	4	67
Buying from the feed shop	38	63.3
**Source of raw materials for homemade (n = 18**) *		
Waste from kitchen	5	27.8
Waste from animals’ product	2	11.1
Buying from markets	13	72.2
Others	1	5.6

* Multiple options, participants can have more than one.

### Participants response on the source of water for fish, training in fish farming and the access to extension services

Concerning water, most of the farms obtained the resource from drilled wells (51.7%), with the frequency of changing water being mostly once in two months (41.7%), mostly basing on water turbidity (56.3%) as shown in Table 3. Additionally, majority of farmers (80%) lacked formal education in aquaculture, while 50% had no access to extension services.

**Table 3:** Water sources, training in aquaculture and access to extension services.

Variable	Frequency (n)	Percent (%)
**Source of water**		
Rain water	1	1.7
Stream	4	6.7
Borehole	4	6.7
Spring water	11	18.3
Municipal water supply	9	15.0
Well	31	51.7
**Frequency of changing water**		
Never changed	12	20
Once after 2 month or more	23	38.3
Once in 2 month or less	25	41.7
Criteria for changing water (n = 48) *		
Water is too turbidly	27	56.3
Depletion of dissolved Oxygen	9	18.8
Fluctuation of pH	1	2.1
Follow a normal schedule for water exchange	13	27.1
Change arbitrarily	9	18.8
**Training on Aquaculture**		
Not trained	48	80
Trained	12	20
**Types of training (n = 12)**		
Aquatic health	5	41.7
Biosecurity and welfare management	8	66.7
Water quality management	9	75
Antimicrobial agents	1	8.3
Environmental monitoring	4	33.3
**Access of the Extension service**		
Yes	30	50
No	30	50
**Frequency of extension services**		
Every week	1	3.3
Once per three months	15	50
Other	14	46.7

* Multiple options, participants can have more than one response.

### Fish farmers response on knowledge on antimicrobial use

**Fig 1:**
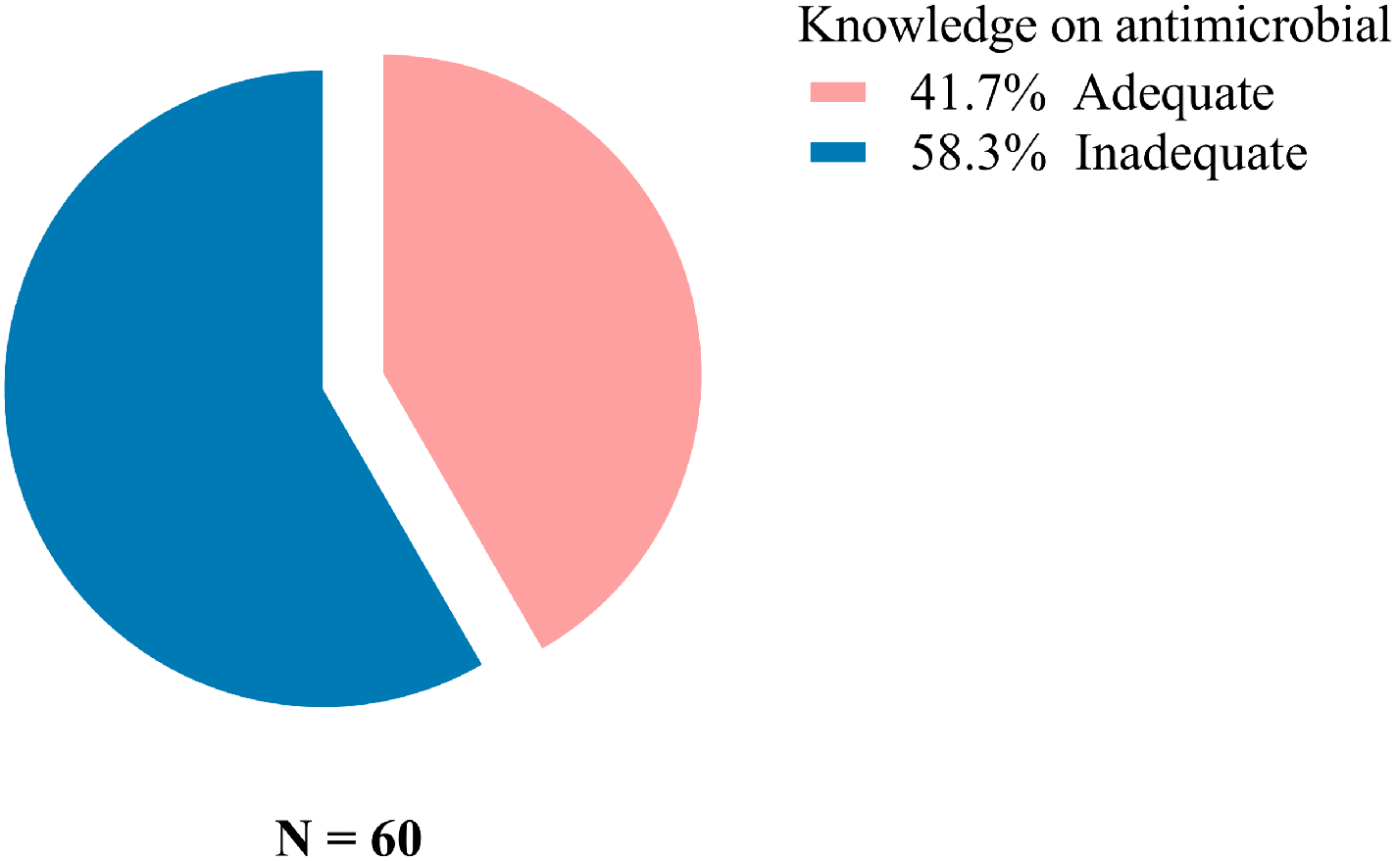
Fish farmer’s response on the adequacy on the knowledge on antimicrobial use.

**Fig 2:**
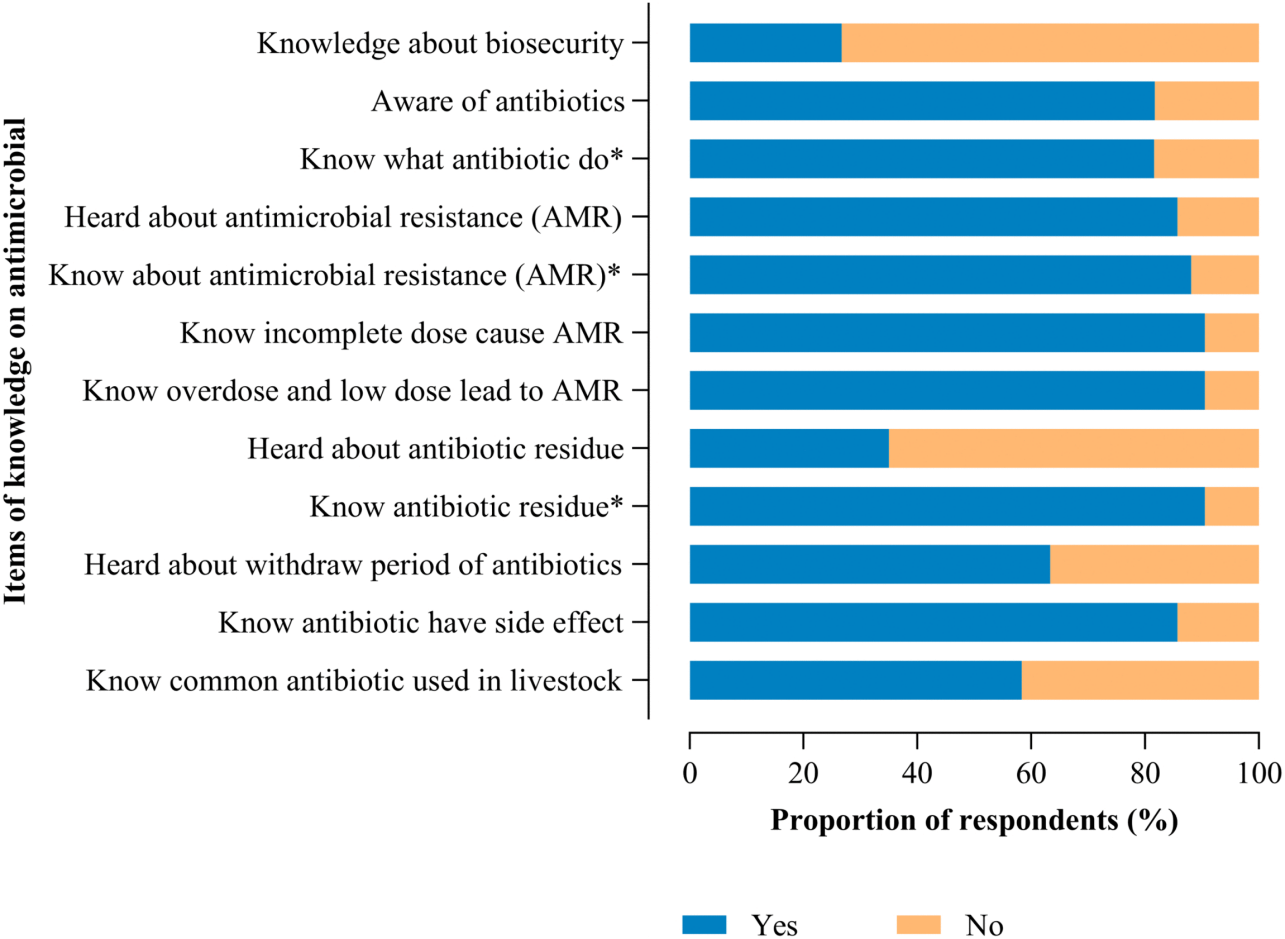
Fish farmers’ response on the knowledge about AMU.

Based on the farmers’ responses about antimicrobial use, only 41.7% were judged to have adequate knowledge (Fig 1). Additionally, most farmers (80%) did not know biosecurity and majority (80%) were knowledgeable on antibiotics. and antimicrobial resistance (93%), and heard about the withdrawal period for antibiotics (63%) as detailed in Fig 2.

### Factors associated with knowledge on antimicrobial agents

As shown in Table 4, factors that related with adequate knowledge of antimicrobial agents were age less than 50 years (24%), formal education beyond the secondary level (53.3%), semi-intensive fish farming (51.4%) and source of water from well (58.1%). Additionally, training on aquaculture, working experience and gender were not associated with knowledge of AMU.

**Table 4:** Factors associated with knowledge on antimicrobial agents.

Variable	Knowledge on antimicrobial		P – value
	Adequate (%)	Inadequate (%)	
**Age group of the owner (years)**			
≤ 50	7 (24.1)	22 (75.9)	0.008
>50	18 (58.1)	13 (41.9)	
**Gender of owner**			
Male	20 (46.5)	23 (53.5)	0.226
Female	5 (29.4)	12 (70.6)	
**Level of education of owner**			
No formal education & primary	1 (6.7)	14 (93.3)	0.001
Secondary & above	24 (53.3)	21 (46.7)	
**Working experience in aquaculture**			
≤ 5 years	13 (34.2)	25 (65.8)	0.124
>5 years	12 (54.5)	10 (45.5)	
**Primary occupation**			
Agricultural activities	6 (26.1)	17 (73.9)	0.054
Non-agricultural activities	19 (51.4)	18 (48.6)	
**Level of management**			
Extensive	0 (0.0)	6 (100)	0.017
Semi intensive	24 (51.1)	23 (48.9)	
Intensive	1 (14.3)	6 (85.7)	
**Source of formulated feed**			
Home made	6 (33.3)	12 (66.7)	0.108
Buying from the feed shop	19 (50.0)	19 (50.0)	
Not using formulated feed	0 (0.00)	4 (100.0)	
**Source of water**			
Well	18 (58.1)	13 (41.9)	0.029
Stream	2 (50.0)	2 (50.0)	
Boreholes	2 (50.0)	2 (50.0)	
Spring water	0 (0.0)	11 (100)	
Municipal water supply	3 (33.3)	6 (67.7)	
Rain water	0 (0.0)	1 (100.0)	
**Frequency of changing water**			
Once per two months or less	12 (48.0)	13 (52.0)	0.404
Once after two months or more	10 (43.5)	13 (56.5)	
Never changing water	3 (25.0)	9 (75.0)	
**Training on Aquaculture**			
Trained	3 (25.0)	9 (75.0)	0.190
Not trained	22(45.8)	26 (54.2)	

**Fig 3:**
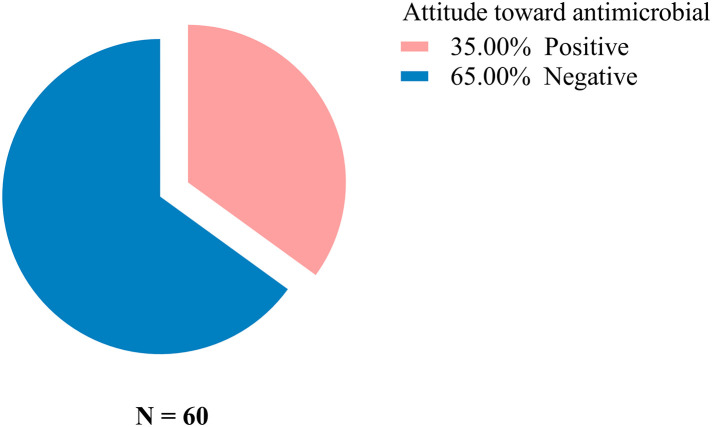
Fish farmer’s response on attitudes regarding antimicrobial use.

**Fig 4:**
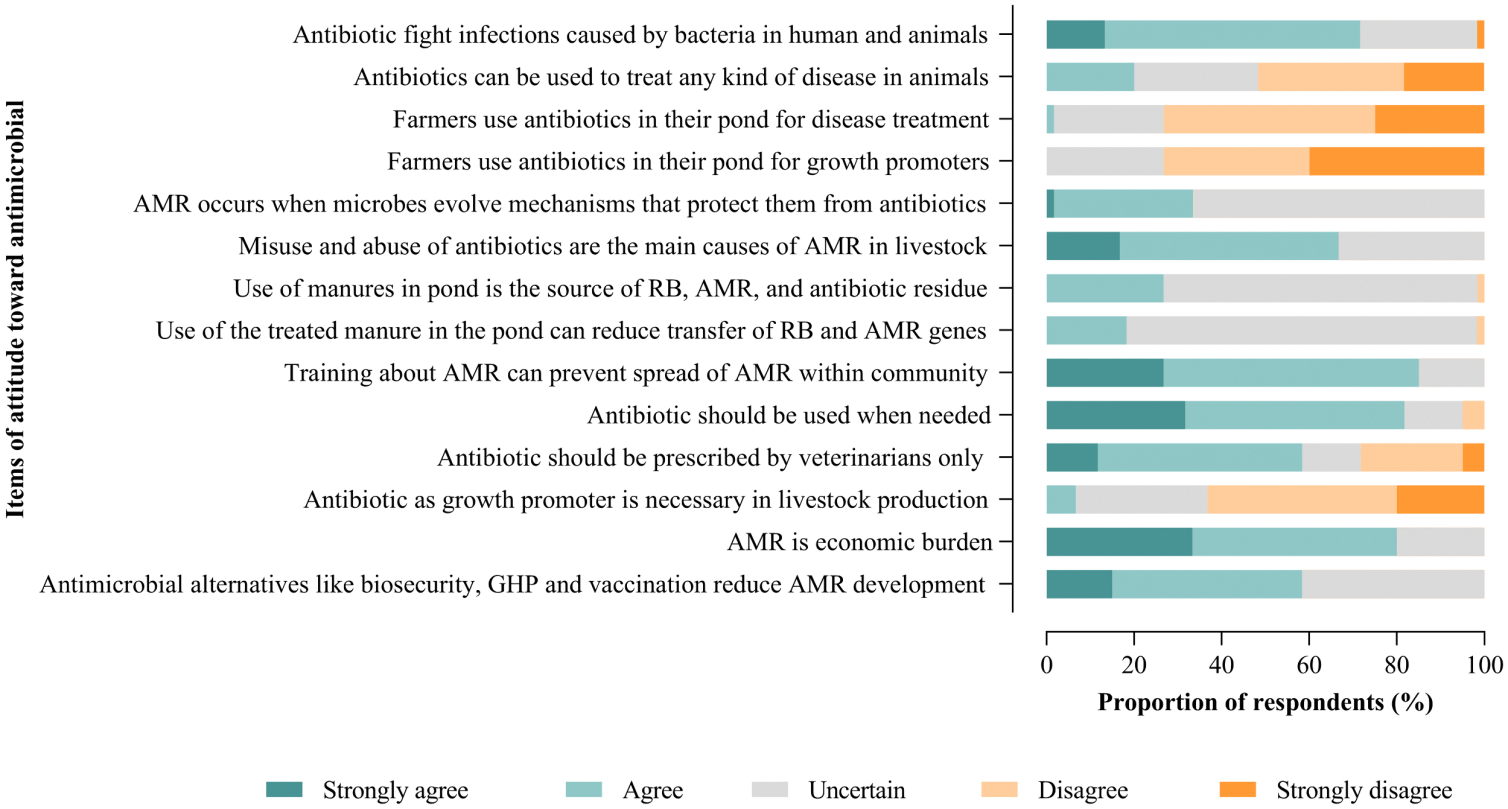
Fish farmers’ specific responses towards their attitudes on AMU.

The attitude of the fish farmers towards AMU shows that only 35% of them had positive attitudes as shown in Fig 3. Additionally, less than 15% strongly agreed that antibiotics could fight infections caused by bacteria, and 15% of farmers indicated that antimicrobial alternatives like biosecurity and vaccination can reduce the development of AMR as shown in Fig 4. Most farmers were uncertain on whether i) treated manure can contribute to AMR (75%) ii) antibiotics should be used for growth promotion or treatment of fish diseases and (30%) iii) pathogens can develop resistance against antimicrobial agents (65%).

### Factors associated with attitude towards antimicrobial agents

The only factors that were associated with positive attitudes towards antimicrobial agents were having a formal education (secondary and above) (44.4%) and knowledge on antimicrobial agents (56%). The attitude towards antimicrobial agents were not associated with age, working experience in aquaculture, primary occupation, and level of management. The detailed information is presented in Table 5.

**Table 5:** Factors associated with attitude toward antimicrobial agents.

Variable	Attitude on antimicrobial		P – value
	Positive (%)	Negative (%)	
**Age group of the owner (years)**			
≤ 50	9 (31.0)	20 (69.0)	0.533
>50	12 (38.7)	19 (61.3)	
**Gender of owner**			
Male	18 (41.9)	25 (58.1)	0.076
Female	3 (17.6)	14 (82.4)	
**Level of education of owner**			
No formal education & primary	1 (6.7)	14 (93.3)	0.008
Secondary & above	20 (44.4)	25 (55.6)	
**Working experience in aquaculture**			
≤ 5 years	11 (28.9)	27 (71.1)	0.196
>5 years	10 (45.5)	12 (54.5)	
**Primary occupation**			
Agricultural activities	6 (26.1)	17 (73.9)	0.254
Non-agricultural activities	15 (40.5)	22 (59.5)	
**Level of management**			
Extensive	1 (16.7)	5 (83.3)	0.576
Semi intensive	17 (36.2)	30 (63.8)	
Intensive	3 (42.9)	4 (57.1)	
**Source of formulated feed**			
Home made	5 (27.8)	13 (72.2)	0.182
Buying from the feed shop	16 (42.1)	22 (57.9)	
Not using formulated feed	0 (0.0)	4 (100.0)	
**Source of water**			
Well	12 (38.7)	19 (61.3)	0.286
Stream	1 (25.0)	3 (75.0)	
Boreholes	2 (50.0)	2 (50.0)	
Spring water	1 (9.1)	10 (90.9)	
Municipal water supply	5 (55.6)	4 (44.4)	
Rain water	0 (0.0)	1 (100)	
**Frequency of changing water**			
Once per two months or less	12 (48.0)	13 (52.0)	0.147
Once after two months or more	7 (30.4)	16 (69.6)	
Never changing water	2 (16.7)	10 (83.3)	
**Training on Aquaculture**			
Trained	5 (41.7)	7 (58.3)	0.588
Not trained	16 (33.0)	32 (66.7)	
**Knowledge**			
Adequate knowledge	14 (56.0)	11 (44.0)	0.004
Inadequate knowledge	7 (20.0)	28 (80.0)	

### Response of the fish farmers on practices associated with fish farming

**Fig 5:**
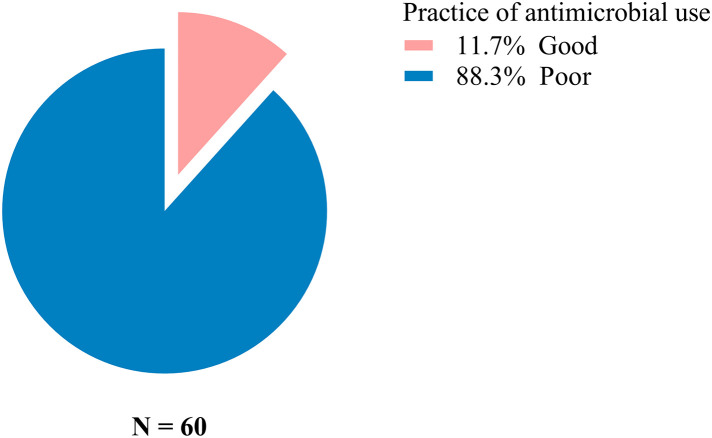
Fish farmer’s response regarding practices of antimicrobial use.

**Fig 6:**
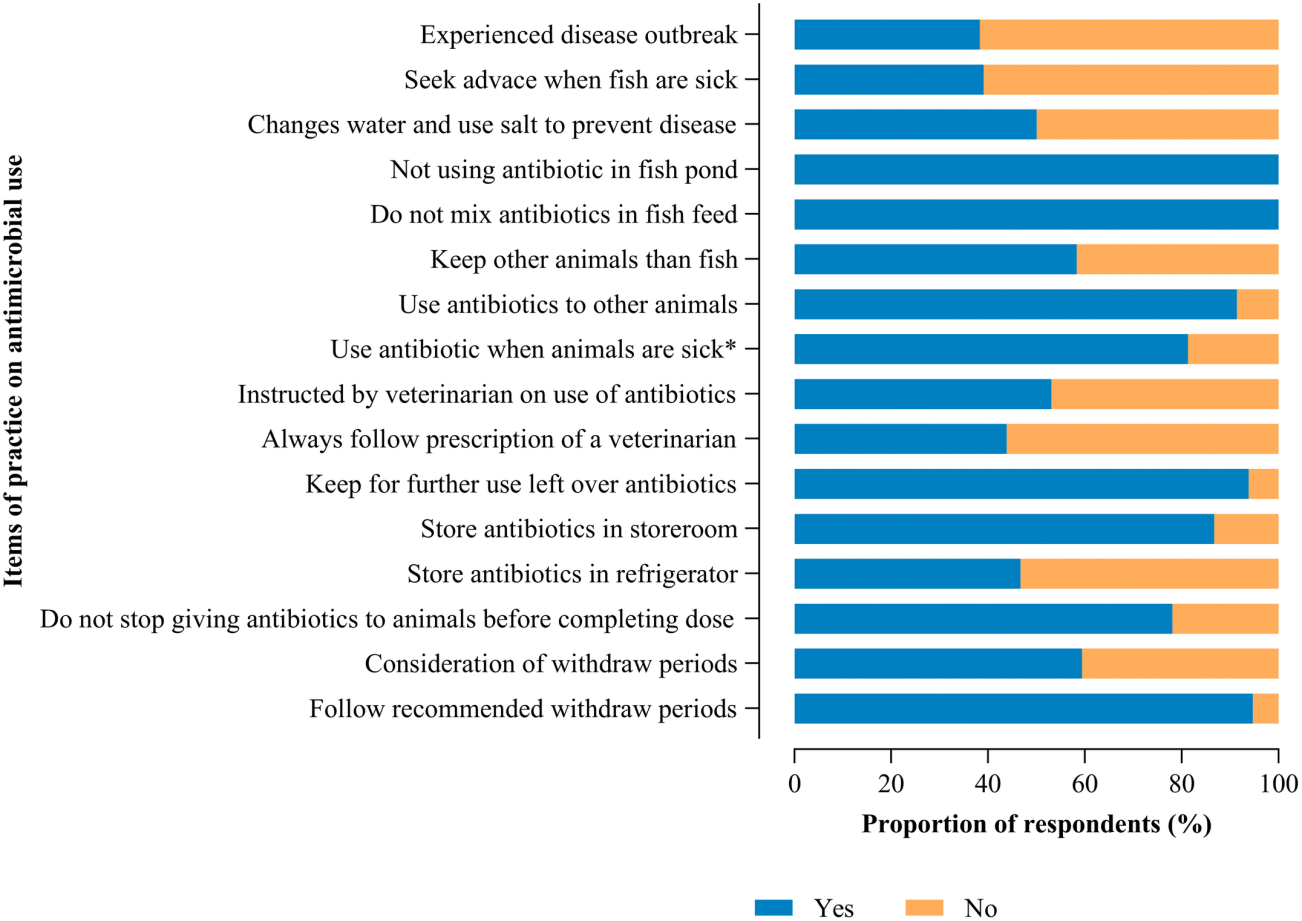
Fish farmers’ specific responses towards AMU practices.

The practices of the fish farmers were judged to have poor practices on antibiotics (88.3%) and more than 90% of farmers indicated they could use antibiotics intended for other animals and keep them for further use in the future. None of the fish farmers indicated using antibiotics either directly or indirectly through fish feed as detailed in Figs 5 and 6.

### Factors associated with practices on antimicrobial use in fish farming

As shown in Table 6, the only factors that were associated with positive practices were adequate knowledge of antimicrobial agents and positive attitudes toward them. Factors such as gender, level of education, primary occupation, source of feeds and water, and training in aquaculture were not associated with practices of antimicrobial use in aquaculture.

**Table 6:** Factors associated with practices antimicrobial use in aquaculture.

Variable	Practice on antimicrobial		P – value
	Good (%)	Poor (%)	
**Age group of the owner (years)**			
≤ 50	1 (3.4)	28 (96.6)	0.055
>50	6 (19.4)	25 (80.6)	
**Gender of owner**			
Male	4 (9.3)	39 (90.7)	0.364
Female	3 (17.6)	14 (82.4)	
**Level of education of owner**			
No formal education & primary	1 (6.7)	14 (93.3)	0.486
Secondary & above	6 (13.3)	39 (86.7)	
**Working experience in aquaculture**			
≤ 5 years	5 (13.2)	33 (86.8)	0.636
>5 years	2 (9.1)	20 (90.9)	
**Primary occupation**			
Agricultural activities	2 (8.7)	21 (91.3)	0.572
Nonagricultural activities	5 (13.5)	32 (86.5)	
**Level of management**			
Extensive	0 (0.0)	6 (100.0)	0.472
Semi intensive	7 (14.9)	40 (85.1)	
Intensive	0 (0.0)	7 (100)	
**Source of formulated feed**			
Home made	1 (5.6)	17 (94.4)	0.645
Buying from the feed shop	6 (15.8)	32 (84.2)	
Not using formulated feed	0 (0.0)	4 (100.0)	
**Source of water**			
Well	5 (16.1)	26 (83.9)	0.541
Stream	0 (0.0)	4 (100.0)	
Boreholes	1 (25.0)	3 (75.0)	
Spring water	0 (0.0)	11 (100.0)	
Municipal water supply	1 (11.1)	8 (88.9)	
Rain water	0 (0.0)	1 (100)	
**Frequency of changing water**			
Once in two months or less	5 (20.0)	20 (80.0)	0.222
Once after two months or more	2 (8.7)	21 (91.3)	
Never changing water	0 (0.0)	12 (100)	
**Training on Aquaculture**			
Trained	1 (8.3)	11 (91.7)	1.000
Not trained	6 (12.5)	42 (87.5)	
**Knowledge**			
Adequate knowledge	6 (24.0)	19 (76.0)	0.017
Inadequate knowledge	1 (2.9)	34 (97.1)	
**Attitude**			
Positive attitude	6 (28.6)	15 (71.4)	0.006
Negative attitude	1 (2.6)	38 (97.4)	

### Fish farmers correlation between knowledge, attitude and practices regarding AMU

Using tailed Spearman Rank correlation coefficients, this study found a statistically significant between knowledge, attitudes and practices and antimicrobial use as shown in Table 7.

**Table 7:** Correlation among farmer’s knowledge, attitude and practices towards AMU.

		Knowledge	Attitude	Practice
Knowledge	Correlation coefficient	1.000	0.784	0.468
	Sig. (2-tailed)	< 0.001	< 0.001	< 0.001
	n	60	60	60
Attitude	Correlation coefficient	0.784	1.000	0.374
	Sig. (2-tailed)	< 0.001	< 0.001	0.004
	n	60	60	60
Practices	Correlation coefficient	0.468	0.374	1.000
	Sig. (2-tailed)	< 0.001	0.004	< 0.001
	n	60	60	60

Key: AMU: Antimicrobial Use, n = number of respondents.

## Discussion

The present study, which involved all fish farmers in Dar es Salaam the largest city and financial hub of Tanzania found that aquaculture is predominantly practiced by males (71.7%), most of whom are over 50 years old (51.7%) and college-educated (38.3%). The majority of farming was semi-intensive and monoculture-based, with a production cycle of 6–8 months, a pattern commonly observed in many African countries [12]. Most farms (61.7%) were individually owned and occupied less than one hectare. Only 13% of the farmers relied entirely on aquaculture as their sole source of income. Unfortunately, the small size and low productivity of these farms hinder smallholders from increasing their incomes and escaping poverty, as observed in a study conducted in Uganda [24].

This investigation did not identify the direct use of antibiotics on any of the farms. However, a related study conducted in the same ponds found that sampled farmed fish tested positive for sulphonamide residues and tetracycline [25]. A likely source of these antibiotics is the use of animal manure primarily poultry droppings for pond fertilization, a practice reported in over a third (38.3%) of the farms. This finding aligns with a study from Ghana, which reported frequent fertilization of ponds with manure from treated animals [26]. Additionally, most fish feeds were purchased from local stores and often lacked proper labeling, making it difficult to determine whether they contained antimicrobial additives. This raises concerns about potential antimicrobial residues, food safety risks, and the broader impact on the pond ecosystem [27].

The study also revealed significant gaps in knowledge, attitudes, and practices related to antimicrobial use and resistance in aquaculture. A large proportion of fish farmers (80%) had not received formal training in aquaculture, 50% lacked access to aquaculture extension services, and 80% were unaware of biosecurity principles. Although 80% were familiar with antibiotics and 93% had heard of antimicrobial resistance (AMR), only 63% had ever received information about antibiotic withdrawal periods, and fewer than 40% were aware of antibiotic residues. These findings are consistent with studies from other African countries [28], but contrast sharply with a study from Turkey, where 96.7% of fish farmers had knowledge of antibiotics, antimicrobial use, AMR, and withdrawal periods [29]. This disparity may be attributed to limited knowledge among Tanzanian fish farmers, a lack of extension services particularly regarding biosecurity and the infrequent use of alternative disease prevention methods such as vaccination.

Regarding attitudes toward antimicrobial use, only 35% of farmers demonstrated a positive attitude. Fewer than 15% strongly agreed that antibiotics are effective against bacterial infections and those alternative strategies, such as biosecurity and vaccination, can reduce the development of AMR. Many farmers were uncertain about several issues, including: i) whether pond fertilization with manure could contribute to AMR, ii) whether antibiotics should be used for growth promotion or solely for disease treatment in fish, and iii) whether pathogens can develop resistance to antimicrobial agents. These findings highlight the urgent need for improved education, targeted support, and the promotion of preventive measures in aquaculture.

Alarmingly, 88.3% of farmers demonstrated poor practices related to antibiotic use. Over 90% reported that they would use antibiotics intended for other animals and store them for future use. Only 50% reported consistently following veterinary prescriptions, indicating a lack of adherence to recommended guidelines. The stocking and misuse of antimicrobials has also been reported in studies from Tanzania, Ghana, and Malaysia [8,30,31], raising concerns about reduced antibiotic effectiveness and the emergence of antibiotic-resistant bacteria in farmed fish threatening both animal and human health [32].

Regarding pond practices, the main water source for most farms (51.7%) was drilled wells. However, water management practices were inconsistent and lacked systematic quality monitoring. Water changes were based on turbidity a subjective visual assessment rather than scientific testing, and pond drainage practices were irregular and poorly managed. In the context of aquaculture development in Tanzania, the Ministry of Livestock and Fisheries (MLF) should establish national water quality standards for aquaculture. The Tanzania Fisheries Research Institute (TAFIRI) should be tasked with monitoring and enforcing these standards. This division of responsibility would ensure that policy-making and regulatory oversight remain under the MLF, while practical implementation and technical support are provided by TAFIRI.

To improve aquaculture practices, training programs on Good Aquaculture Practices (GAqP) should be implemented by extension officers from the MLF and local governments, in collaboration with TAFIRI, with a focus on proper pond preparation techniques. Additionally, the Tanzania Bureau of Standards (TBS) should ensure compliance with these standards, and the National Fish Quality Control Laboratory (NFQCL) should establish surveillance systems to monitor antibiotic use and resistance patterns. Notably, the National Fisheries and Aquaculture Research Agenda (2020–2025) [33] is approaching completion, providing a critical opportunity for review and renewal. The next agenda should address the gaps and limitations of its predecessor, with a strong emphasis on antimicrobial use, resistance, and residue monitoring in fisheries and aquaculture. Priority should be placed on strategic and efficient resource allocation, fostering multisectoral collaboration among government agencies, research institutions, industry, and communities, and ensuring research outputs are effectively translated into practical, scalable interventions that drive sustainable sector growth and safeguard public health.

Tanzania has already established a legal and regulatory framework for aquaculture development, including the National Fisheries Policy (2015) [34] and the Fisheries (Aquaculture) Regulations (2024) [35]. The Tanzania Food and Drugs Act (2003) [36] governs the sale of veterinary drugs. However, enforcement mechanisms remain weak or absent. Recently, the country developed a National Action Plan on Antimicrobial Resistance (NAP-AMR, 2023–2028) [37], which outlines a multisectoral approach to addressing AMR across human, animal, and environmental health sectors including aquaculture. Implementation of this plan, however, faces several challenges: limited financial and human resources, a lack of context-specific tools, inadequate coordination and collaboration, limited farmer knowledge, and weak surveillance systems.

In summary, we recommend several interventions: i) Raise awareness among fish farmers about the prudent use of antibiotics, ii) Strengthen extension services as outlined in the aquaculture thematic areas of the Fisheries Sector Master Plan of 2021/22–2036/37 (URT 2021) [38], ensuring that training is context-specific and guided by the Ecosystem Approach to Aquaculture, iii) Establish a functional surveillance system for monitoring antimicrobial use (AMU) and resistance (AMR). The Ministry of Livestock and Fisheries, through its Department of Aquaculture, should develop cost-effective, farmer-to-farmer extension models and clear guidelines for monitoring AMU, AMR, and disease management in fish farming. This initiative should involve key institutions, including universities, Livestock Training Agencies, the Tanzania Livestock Research Institute, the Tanzania Fisheries Research Institute (TAFIRI), and the Fisheries Education Training Authority (FETA). Finally, to improve access to information and promote peer learning, we propose the formation of small cooperative groups under the Aquaculture Association of Tanzania (AAT), and the use of social media platforms such as WhatsApp, Facebook, and X (formerly Twitter) for networking and knowledge sharing.

## Conclusion

The current investigation’s findings establish fundamental evidence about the knowledge, attitudes, and practices (KAP) of fish farmers in low-income nations such as Tanzania. These findings provide valuable insights for establishing interventions and policies related to the antimicrobial use (AMU) and antimicrobial resistance (AMR) in the country. This study provides valuable insights for the sustainable growth of aquaculture in Tanzania and highlights the need for strategic interventions to address current challenges. Strengthening aquaculture extension services is essential, with programs focused on delivering practical, context-specific solutions and involving farmers and field extension workers in jointly identifying research needs and designing solutions. Establishing a robust fish disease surveillance system is critical for monitoring and managing both existing and emerging threats, with the Ministry of Livestock and Fisheries (MLF) developing clear guidelines for tracking antimicrobial use (AMU), antimicrobial resistance (AMR), and fish health, and integrating these efforts with extension and research initiatives. Collaborative research among government agencies, the private sector, academia, and research institutions should be enhanced, ensuring that findings are translated into actionable recommendations for farmers. In addition, the Aquaculture Association of Tanzania (AAT) should promote the formation of small cooperative groups to facilitate knowledge sharing, improve access to information, and strengthen collective problem-solving. Together, these measures will improve productivity, resilience, and sustainability in Tanzania’s aquaculture sector while mitigating AMU and AMR risks.

## Supporting information

S1 File Data Batch 1. (XLSX)

S2 File Data Batch 2. (XLSX)
